# Role of Mitochondria and Lysosomes in the Selective Cytotoxicity of Cold Atmospheric Plasma on Retinoblastoma Cells

**DOI:** 10.22037/ijpr.2020.114165.14703

**Published:** 2020

**Authors:** Ghazaleh Tahmasebi, Esmaeil Eslami, Parvaneh Naserzadeh, Enayatollah Seydi, Jalal Pourahmad

**Affiliations:** a *Department of Atomic/Molecular Physics, Faculty of Physics, Iran University of Science and Technology, Tehran, Iran. *; b *Department of Pharmacology and Toxicology, Faculty of Pharmacy, Shahid Beheshti University of Medical Sciences, Tehran, Iran. *; c *Nanomedicine and Tissue Engineering Research Center, Shahid Beheshti University of Medical Sciences, Tehran, Iran. *; d *Department of Occupational Health and Safety Engineering, School of Health, Alborz University of Medical Sciences, Karaj, Iran. *; e *Research Center for Health, Safety and Environment, Alborz University of Medical Sciences, Karaj, Iran.*

**Keywords:** Retinoblastoma, Mitochondria, Cold plasma, Reactive oxygen species

## Abstract

Retinoblastoma (RB) is a common malignancy in childhood, with an incidence of 1 per 20,000 live births. Several approaches such as chemotherapy, laser, and radiotherapy have been used for the treatment of RB. However, the effectiveness of these methods is not sufficient and the mechanisms involved in the pathogenesis of the disease are not well understood. The disruption of the apoptotic process is considered as one of the mechanisms involved in the pathogenesis of RB. This study was designed to examine the *in-vitro *selective toxicity of cold atmospheric plasma (CAP) on RB cells’ mitochondria and lysosomes. The results showed that CAP decreased cell viability and GSH content and also increased caspase-3 activity and lipid peroxidation (LPO) in cancerous ocular cells isolated from the rat model of RB compared to the normal rat ocular cells. Furthermore, results demonstrated that CAP significantly increased ROS generation, mitochondrial membrane potential (MMP) collapse, mitochondrial swelling, and cytochrome c release only in cancerous rat ocular mitochondria but not the normal rat ocular mitochondria. Furthermore, our results demonstrated that CAP significantly increased the lysosomal damage only in the cancer group. Altogether, the results of the study showed that CAP could selectively induce apoptosis on RB mitochondria. CAP may therefore be considered as a promising candidate for further *in-vivo* and clinical researches to reach a new anti- RB drug.

## Introduction

Retinoblastoma (RB) is one of the common cancers in the intraocular that usually occurs in children (less than five years) (1-3). The statistics show that the worldwide incidence of this disease is nearly 1 case in every 20,000 live births, and the survival rate in the less developed countries is very low. Many approaches such as chemotherapy, frozen method, and eyeball enucleation have been used for the treatment of RB, but their effectiveness is low (1, 3). Therefore, the use of new approaches can help RB therapy. Also, an approach should be selected that can kill cancer cells and have no effect on normal cells. Apoptosis inhibition is one of the important mechanisms that play an crucial role in the occurrence of RB, in which the expression of anti-apoptotic proteins is increased in comparison with pro-apoptotic proteins (1, 5). The induction of apoptosis can be considered as one of the treatment approaches for this disease.

*In-vivo* and *in-vitro* studies have shown that cold atmospheric plasma (CAP) has been used to treat a variety of cancers such as colon, prostate (8-11). In recent years, plasma has attracted many researchers due to anticancer effects and has led to the emergence of a new field in medical science termed plasma oncology (12, 13). One of the most important mechanisms by which CAP causes death in cancer cells is through the increase in the generation of the reactive oxygen species (ROS) (9, 10, 12, and 14). Furthermore, CAP can induce an apoptotic process in cancerous cells through ROS generation. However, the molecular mechanism of CAP has not yet been well known (15-17).

Apoptosis is considered to be one of the main pathways of cell death. Apoptosis occurs either through the Intrinsic/mitochondrial-mediated pathway or external/ death receptor-mediated pathway. In the internal pathway, events such as the activation of caspases and pro-apoptotic proteins (Bcl-2 family members) occur, which subsequently lead to changes in the outer mitochondrial membrane, cytochrome c release, and the formation of the apoptosome. The extrinsic pathway initiated with the activation of death receptors (FasL, TNFα, TRAIL receptors). Furthermore, biochemical changes in apoptosis include activation of caspases, DNA and protein breakdown, and Membrane changes (18-21). 

ROS play a double role in cells. ROS play a role in critical physiological processes, such as cell growth and cell proliferation (at low to moderate concentrations), as well as cell death (at high concentrations) (14, 17). Research has shown that the level of ROS in cancer cells is slightly higher than normal cells. Moreover, a cancer cell is more sensitive to the increase of ROS generation than its corresponding normal cell (22, 23). On the other hand, the increase in the level of ROS in cancer cells is known as one of the most promising approaches in cancer therapy (24). Therefore, the use of compounds that can increase ROS in cancer cells can help cancer therapy. Mitochondria are known as an important source of intracellular ROS (24, 25). The statistics show that about 90% of intracellular ROS are generated by mitochondria (24). Therefore, targeting mitochondria and increasing the generation of ROS in these organelles can kill cancer cells. Therefore, this study was designed to investigate the selective cytotoxic effects of CAP on RB mitochondria via ROS-mediated apoptosis signaling. 

## Experimental

*Chemical*

2,7-Dichlorofluorescein diacetate (DCHF-DA), rhodamine 123 (Rh 123), and caspase-3 assay kit were purchased from Sigma Chemical Co (St. Louis, MO), and cytochrome c assay kit (was purchased from R and D Systems, Inc. (Minneapolis, MN). In addition, all other chemicals were of the highest commercial grade available.

*Animals*

The RB rat model and normal rat were purchased from the Institute Pasteur (Tehran, Iran). Rats were kept in a temperature-controlled environment on a 12:12 h light/dark cycle. All investigations were performed according to the guidelines of ethical standards and the Institutional Animal Care and Use Committee (IACUC) of Shahid Beheshti University of Medical Sciences in Tehran, Iran.

*Cells and mitochondria isolation*

Rats with RB were euthanized. Then, one eye was removed and 1 × 10^6^ cells were dispersed and maintained in culture medium (RPMI 1640 medium containing 11.1 mmol/L glucose, 50 μmol/L β-mercaptoethanol, 1.0 mmol/L sodium pyruvate, 2.0 mmol/L L-glutamine, 100 U/mL penicillin, 100 μg/mL streptomycin, and 10% fetal bovine serum (Gibco, MD, USA)). For mitochondrial isolation, rat retinoblastoma cells were washed twice in mitochondrial extraction buffer (HEPES 10 mM, mannitol 200 mM, sucrose 70 mM, EGTA 1 mM, and pH 7.5) and then were resuspended in 10 vol extraction buffer (Mitochondrial Isolation Kit; Sigma). Rat retinoblastoma cells were homogenized and then nuclei and intact cells were removed via centrifugation at 600 *×g *for 5 min. In the next step, the supernatant was centrifuged at 11,000 ×*g *for 10 min. Then, the pellet was resuspended in a 10 vol extraction buffer, and centrifugation was performed in two steps (600*×g *and 11,000 ×*g*, respectively). Finally, the mitochondrial fraction (final pellet) was resuspended in assay buffer (MOPS 20 mM, KCl 110 mM, ATP 10 mM, MgCl_2_10 mM, sodium succinate 10 mM, EGTA 1 mM, and pH 7.5) and was stored for short periods on ice until the further investigation (26, 27). 

Furthermore, for the normalization of the mitochondrial samples in all experiments, the same mitochondrial protein concentration for each sample (0.5 mg mitochondrial protein/mL) was used. In this study, mitochondrial function following their extraction was checked by the MTT test (for evaluation of mitochondrial function, mitochondrial complex II activity was determined). The mitochondrial membrane integrity was checked by the cytochrome c oxidase (complex IV) assay kit following their initial extraction. 

*Cold Atmospheric Plasma (CAP) treatment*

The plasma device used in this study is an atmospheric pressure plasma argon plasma jet. The device made at the University of Iran Science and Technology includes a 1.5 mm diameter central copper pin electrode inside a glass tube (Pyrex) connected to a high voltage resonant transformer and outer aluminum electrode wrapped around it. The inner and outer diameter of the glass tube was 5 mm and 8 mm, respectively. The output voltage and the frequency was 4 kV and ~ 40 kHz, respectively. In this work, the feeding gas was 99.999% pure Argon (Ar) with a 10 L/min gas flow rate. The electrical circuit includes a power supply, an electrode (aluminum and copper), interface wires, and an oscilloscope. The electrodes are centered and axially symmetrical. The central HV electrode is a 1.5 mm diameter copper rod and a cylindrical and aluminum-coated electrode. In the production and stability of the plasma created by the electric field, the production of secondary electrons plays an essential role. Secondary electrons, in fact, are electrons emitted from the metal cathode level, which are formed by the impact of high-energy ions and atoms. This process is characterized by a parameter called the secondary electron emission coefficient and the cathode genus used in this parameter. According to studies, the metal is suitable for use as a cathode, which, in addition to the high electron emission factor, is also low.

For this reason, aluminum has been used in this study because of its high secondary electron emission coefficient and the low rate of sputtering as a cathode. Electrode HV was copper. Argon gas is transferred to the plasma jet cylinder from the capsule by a 6 mm pneumatic hose, but before entering the cylinder, the gas is entered into the flowmeter and controlled by the flowmeter to control the flow and velocity. In this study, a KT800-6 glass flowmeter with the ability to measure 100-1000 liters per hour with a precision of 40 liters per hour was used to control and measure the flow rate of gas. Also, the distance between the two electrodes and the pressure was 6.5 mm and 760 Torr, respectively. In order to have a stable plasma, an alternating sinusoidal voltage source, together with a transformer, was used as an amplifier. To use plasma for isolated mitochondria, it was necessary to test the power supply at different voltages and frequencies. Finally, the best mode for producing an ideal and uniform plasma was the output voltage of 4 kV and the frequency of 40 kHz, which was measured with an oscilloscope. Also, the gas pressure used in experiments at 600 L/h was about 10 liters per minute. By increasing gas pressure, the length of the plasma can be changed. Also, the nozzle distance from the 96-well plate was about 12 mm. This distance is almost ideal because as the distance increases, the plasma effect decreases, and also by decreasing the distance, in addition to the gas pressure causing the particles to disperse, also causes the arc, especially at a distance of less than 8 mm and this is due to the conductivity of the sample and the increase of the instantaneous electric current. The discharge is ignited inside a dielectric tube within a gap between two electrodes. The inner and outer diameter of the dielectric tube was 5 mm and 8 mm, respectively.

**Figure 1:**
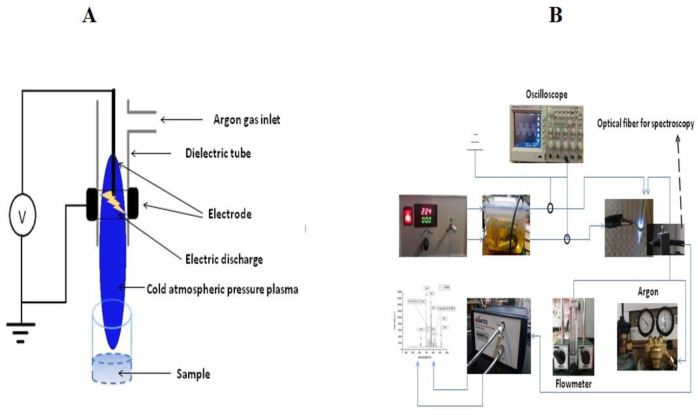
(A-B) Cold atmospheric plasma (CAP) treatment scheme

The distance from the tip of the nozzle of the plasma jet and the sample was exactly 20.25 mm. The time durations for plasma treatment were 30, 60, and 120 sec (Figures 1A and 1B). Our time for mitochondrial retinoblastoma treatment was 30, 60, and 120 sec. According to Cheng *et al.*, We calculated the dose of plasma from 𝐷~𝑄∗𝑉∗T , where D is the entire “plasma dosage” applied to mitochondria; Q is the flow rate of the feeding gas, V is the output voltage and T is the treatment time (14). The scheme and characteristics of the CAP are shown in Figures 1A and 1B and Table 1, respectively. Finally, the dose of 200, 400, 800, 1200, 2400, 2800, 3600, and 4800 a.u. of CAP were used to evaluate succinate dehydrogenase (SDH) activity assay. The dose used in the other experiments were selected based on the SDH activity assay. 

*Mitochondrial assay*

*Evaluation of Succinate Dehydrogenase (SDH) activity*

In this study, MTT (3-[4,5-dimethylthiazol-2-yl]-2,5-diphenyltetrazolium bromide) probe was used to assess the effects of CAP (200, 400, 800, 1200, 2400, 2800, 3600 and 4800 a.u.) on mitochondrial SDH activity. The mitochondria were isolated and exposed to various doses of the CAP (200, 400, 800, 1200, 2400, 2800, 3600, and 4800 a.u.) for 1 h at 37 °C. To assess the mitochondrial SDH activity, the MTT dye was added to the medium and incubation was done for 30 min (37 °C). Dimethyl sulfoxide (DMSO) shas been used to dissolve formazan crystals. Finally, the absorbance was assayed using an ELISA reader (Tecan, Rainbow Thermo, Austria) at 570 nm (28).

*ROS level assay*

The 2′, 7′-dichlorodihydrofluorescein diacetate (DCFH-DA) probe was used to measure the effects of CAP at the dose of 1200, 2400, and 4800 a.u for on ROS generation. At first, the mitochondria isolated from retinoblastoma and normal groups were exposed to cold plasma (1200, 2400, and 4800 a.u) for 1 h at 37 °C. In the following, the DCFH-DA probe was added to the medium and incubated at 37 °C for 1 h. Then, the fluorescence intensity (DCF) was measured using a fluorescence spectrophotometer (Shimadzu RF5000U) (λ_ex_ = 495 nm, and λ_em_= 530 nm) (29).

*Mitochondrial Membrane Potential (MMP) assay*

The rhodamine 123 (Rh 123) probe was used to evaluate the effects of cold plasma (1200, 2400, and 4800 a.u) on MMP collapse. At first, the mitochondria isolated from retinoblastoma and normal groups were exposed to CAP (1200, 2400, and 4800 a.u) for 1 h at 37 °C. In the following, the DCFH-DA probe was added to the medium and incubated at 37 °C for 1 h. Then, the fluorescence intensity (Rh 123) was measured using a fluorescence spectrophotometer (Shimadzu RF5000U) (λ_ex_ = 470 nm, and λ_em_= 540 nm) (30).

*Mitochondrial swelling assay*

Briefly, isolated mitochondria from retinoblastoma and normal groups were suspended in swelling assay buffer. In the next step, the mitochondrial suspension was incubated with 1200, 2400, and 4800 a.u of CAP for 1 h. Finally, absorbance was measured at 540 nm using an ELISA reader (Tecan, Rainbow Thermo, Austria). The decrease in absorbance at 540 nm indicates a swelling in the mitochondria.

*Cytochrome c release*

Briefly, the Quantikine Rat/Mouse Cytochrome c Immunoassay kit (R and D Systems, Inc., Minneapolis, MN, USA) was used for the determination of cytochrome c release. In this test, a specific monoclonal antibody was pre-coated onto the micro-plate, and then conjugate (75 mL), standard solution, and positive control (50 mL) were added to each well of the micro-plate. Then, we added the protein (1 mg) from each supernatant fraction to the sample wells. In the next step, standards, controls, and samples were added to two wells of the micro-plate. Then, the substrate solution (100 mL) was added to each well and incubated for 30 min, and the stop solution (100 mL) was then added to each well. Finally, the optical density was evaluated at a wavelength of 450 nm.

*Cellular assay*

*Cell viability assay*

MTT (3-[4,5-dimethylthiazol-2-yl]-2,5-diphenyltetrazolium bromide) probe was used to assess the effects of CAP (250, 500, 1000, 1200, 2000, 3000, and 4000 a.u.) on RB cells viability. The RB cells were isolated and exposed to various doses of the CAP (250, 500, 1000, 1200, 2000, 3000, and 4000 a.u.) and maintained in a culture medium (RPMI 1640). To assess the RB cells viability, the MTT dye was added to the medium and incubation was done for 30 min (37 °C). Dimethyl sulfoxide (DMSO) has been used to dissolve formazan crystals. Finally, the absorbance was assayed using an ELISA reader (Tecan, Rainbow Thermo, Austria) at 570 nm (32).

*Lipid Peroxidation (LPO) content assay*

The Malondialdehyde (MDA) level was measured to assess lipid peroxidation (LPO). The level of MDA formed in each of the samples was evaluated by measuring the absorbance of the supernatant at 532 nm with an ELISA reader (Tecan, Rainbow Thermo, Austria). MDA content was expressed as µg/mg protein (33).

*Glutathione (GSH) content assay*

For this test, the 0.5 mL of TCA 10% was added to the cell and then centrifuged at 11,000 RPM for 2 min; for GSH and glutathione disulfide (GSSG) assay, 0.5 mL of supernatant were diluted by the addition of 4.5 mL phosphate-EDTA buffer. One-hundred microliter of diluted supernatant was added to 2.8 mL phosphate-EDTA buffer and 100 µL of the OPT solution. After incubation for 15 min at room temperature, each sample was measured for GSH and GSSG level in quartz cuvettes using the Shimadzu RF-5000 U fluorescence spectrophotometer (λ_ex_ = 350 nm, and λ_em_ = 420 nm).

*Caspase-3 activity assay*

Caspase-3 activity was evaluated using the Sigma Caspase-3 assay kit (Sigma-Aldrich, Taufkirchen, Germany). This assay is based on the hydrolysis of substrate peptide (Ac-DEVD-pNA) by caspase-3. Then, the released p-nitroaniline has a high absorbance at 405 nm. Finally, the concentration of the p-nitroaniline (mM) released from the substrate is evaluated from the absorbance wavelength at 405 nm (34).

*Lysosomal membrane integrity assay*

In this study, acridine orange, a fluorescent dye, was used to evaluate lysosomal membrane damage via CAP (1000, 2000, and 4000 a.u) in the RB cells (10^6^ cells). After the exposure to CAP for 1 h at 37 °C, acridine orange redistribution in the cell suspension (10^6^ cells) was assayed using a Shimadzu RF5000U fluorescence spectrophotometer (λ_ex_ = 495 nm and λ_em_ = 530 nm) (35, 36).

*Statistical analysis*

The data were shown as the means ± standard deviation (SD). The one-way ANOVA analysis (GraphPad Prism software, version 5) was used to determine differences between the mean values. *P* < 0.05 was consid­ered to display a statistically significant difference. All experiments were analyzed using one-way analysis of variance (ANOVA) followed by the Tukey-test.

## Results

*Mitochondrial assay*

*CAP declined SDH activity*

**Figure 2:**
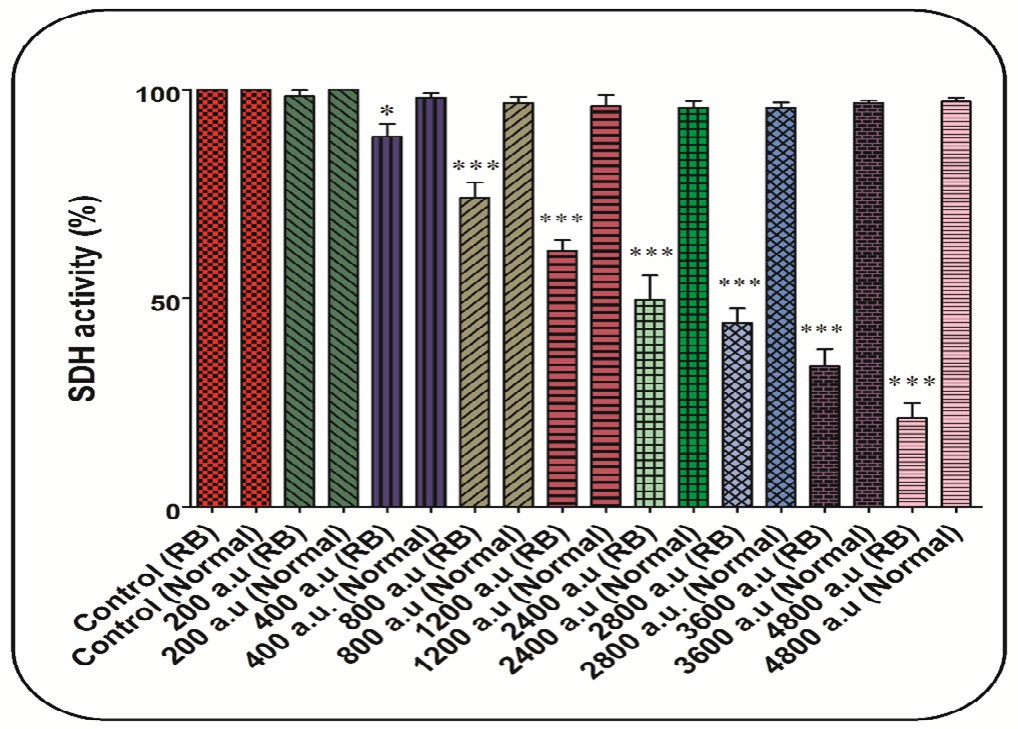
SDH activity assay. The effect of CAP (200, 400, 800, 1200, 2400, 2800, 3600, and 4800 a.u.) on SDH activity in the mitochondria isolated from the normal and retinoblastoma groups. Data are shown as mean ± SD (n = 3). ^*^ and ^***^ show a significant difference in comparison with the corresponding control (*P* < 0.05 and *P* < 0.001, respectively

As shown in Figure 2, the CAP at all doses (200, 400, 800, 1200, 2400, 2800, 3600, and 4800 a.u.) has been able to decrease the SDH activity significantly. In comparison, this change has not been reported in the normal group (Figure 2). In this study, the dose of 2400 a.u. is considered as IC_50_ (Figure 2).

*CAP induced ROS generation*

**Figure 3:**
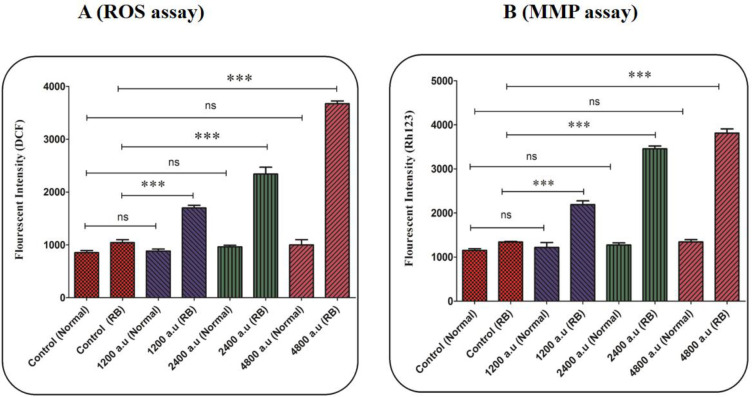
(A) ROS level assay. The effect of CAP (1200, 2400, and 4800 a.u.) on ROS in the mitochondria isolated from the normal and retinoblastoma groups. Data are shown as mean ± SD (n = 3). ^***^ show a significant difference in comparison with the corresponding control (*P* < 0.001). (B) Mitochondrial membrane potential (MMP) assay. The effect of CAP (1200, 2400, and 4800 a.u.) on MMP collapse in the mitochondria isolated from the normal and retinoblastoma. Data are shown as mean ± SD (n = 3). ^***^ show a significant difference in comparison with the corresponding control (*P *< 0. 001).

In the retinoblastoma group, generation of mitochondrial ROS significantly increased after 1 h of incubation with CAP at the dose of 1200, 2400, and 4800 a.u. (Figure 3A). In the normal group, exposure to CAP (1200, 2400, and 4800 a.u.) has not changed the level of ROS (Figure 3A).

*CAP induced MMP collapse*

As is shown in Figure 3B, CAP at all applied doses (1200, 2400, and 4800 a.u.) has been able to collapse the MMP. Whereas the MMP collapse has not been observed in the mitochondria isolated from the normal group (Figure 3B). An increase in fluorescence intensity using Rh 123 probe indicates a collapse in the MMP.

*CAP induced swelling in mitochondria*

**Figure 4:**
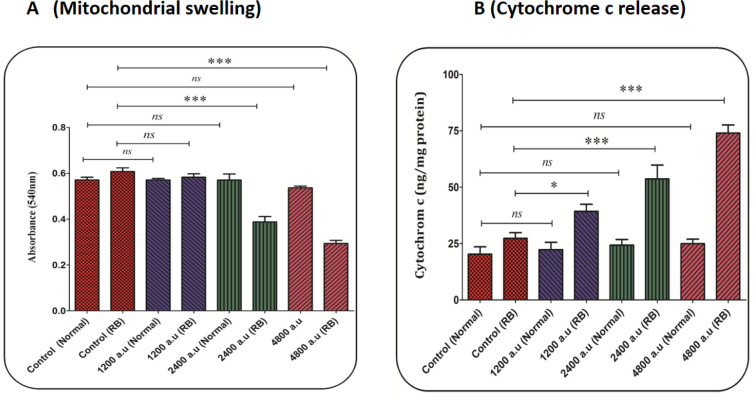
(A) Mitochondrial swelling assay. The effect of CAP (1200, 2400, and 4800 a.u.) on mitochondrial swelling in the mitochondria isolated from the normal and retinoblastoma groups. Data are shown as mean ± SD (n = 3). ^***^ show a significant difference in comparison with the corresponding control (*P *< 0. 001). (B) Cytochrome c release assay. The amount of expelled cytochrome c from the mitochondrial fraction into the suspension buffer was determined using a rat/mouse cytochrome c ELISA kit. Data are presented as mean ± SD (n = 3). ^*^ and ^***^ show a significant difference in comparison with the corresponding control (*P *< 0.05 and *P *< 0.001, respectively)

The decrease in absorbance at 540 nm indicates a swelling in the mitochondria. Accordingly, the results displayed that CAP at a dose of 2400 and 4800 a.u. induced swelling in mitochondria isolated from the retinoblastoma group (Figure 4A). But, CAP at a dose of 1200 a.u. did not affect mitochondrial swelling in the retinoblastoma group. In the normal group, changes in mitochondrial swelling due to exposure to different doses of CAP have not been reported (Figure 4A).

*CAP increased cytochrome c release*

As shown in Figure 4B, the CAP at all doses (1200, 2400, and 4800 a.u.) has led to the release of cytochrome c from the mitochondria in the retinoblastoma group. However, this change has not been reported in the normal group (Figure 4B).

*Cellular assay*

*CAP decreased RB cell viability*

**Figure 5.**
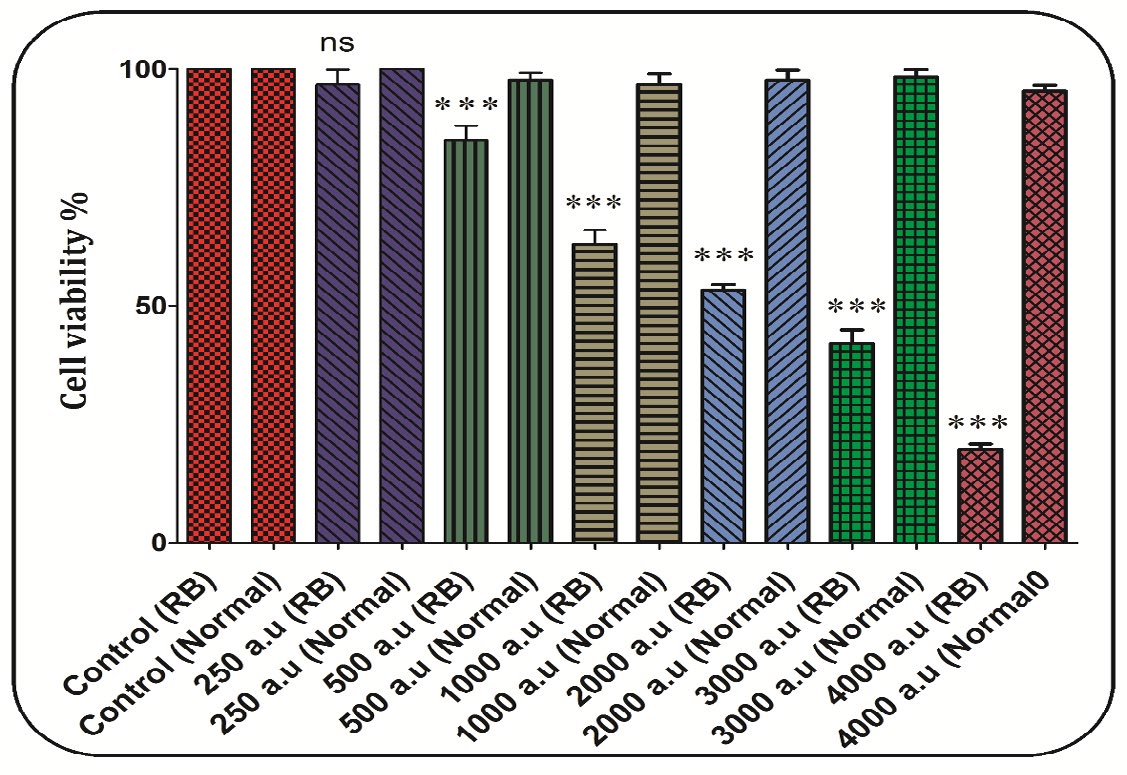
The effect of CAP (250, 500, 1000, 2000, 3000, and 4000 a.u.) on cell viability in the retinoblastoma and normal groups. Data are shown as mean ± SD (n = 3). ^***^ show a significant difference in comparison with the corresponding control (*P* < 0.001. (

In the retinoblastoma group, cell viability significantly decreased after incubation with CAP at the dose of 500, 1000, 1200, 2000, 3000, and 4000 a.u. but not 250 a.u (Figure 5). In the normal group, exposure to CAP (250, 500, 1000, 1200, 2000, 3000, and 4000 a.u. has not decreased the RB cell viability (Figure 5). Also, the dose of 2000 a.u. is considered as IC_50_ (Figure 5).

*CAP increased LPO content*

**Figure 6:**
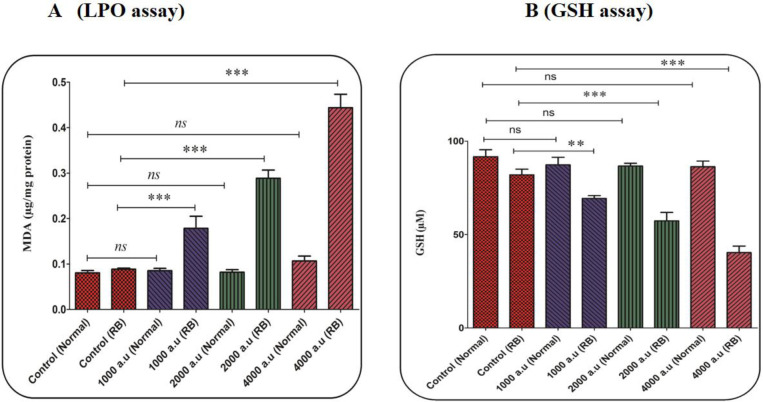
(A) Lipid peroxidation (LPO) content assay. The effect of CAP (1000, 2000, and 4000 a.u.) on LPO content in the mitochondria isolated from the normal and retinoblastoma groups. Data are shown as mean ± SD (n = 3). ^***^ show a significant difference in comparison with the corresponding control (*P* < 0. 001). (B) Glutathione (GSH) content assay. The effect of CAP (1000, 2000, and 4000 a.u.) on GSH content in the mitochondria isolated from the normal and retinoblastoma groups. Data are shown as mean ± SD (n = 3). ^**^ and ^***^ show a significant difference in comparison with the corresponding control (*P* < 0.01 and *P* < 0.001, respectively)

In this study, malondialdehyde (MDA) has been evaluated as an important indicator of LPO. In the retinoblastoma group, the results showed that the CAP increased the LPO content in a dose-dependent pattern (Figure 6A). In the normal group, this change has not been reported (Figure 6A).

*CAP decreased GSH content*

In the antioxidant system, GSH is considered as one of the most important factors. Also, an imbalance between the free radical generation and antioxidant system (such as GSH) in cells is associated with alterations in the homeostasis redox. The decrease in the GSH level supports the collapse in the redox state of the cell. As shown in Figure 6B, the level of GSH considerably decreased only in the retinoblastoma group after exposure to CAP (1000, 2000, and 4000 a.u.).

*CAP induced Caspase-3 activation*

**Figure 7:**
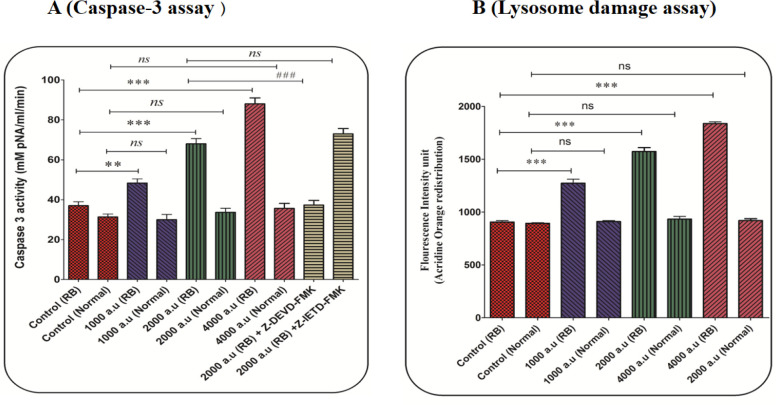
(A) Caspase-3 activity assay. Caspase-3 activity was evaluated using the Sigma Caspase-3 assay kit. The effect of CAP (1000, 2000, and 4000 a.u.) on caspase-3 activity in the retinoblastoma and normal groups. Data are presented as mean ± SD (n = 3). ^***^ show a significant difference in comparison with the corresponding control (*P *< 0.001(. ^###^ show a significant difference between 2000 a.u with 2000 a.u plus z-DEVD (*P *< 0.001(. (B) Lysosomal damage assay. The effect of CAP (1000, 2000, and 4000 a.u.) on lysosomal damage in the normal and retinoblastoma groups. Data are shown as mean ± SD (n = 3). ^***^ show a significant difference in comparison with the corresponding control (*P *< 0.001)

The concentration of the p-nitroaniline released at 405 nm was used for caspase-3 activity. In the retinoblastoma group, activation of caspase-3 significantly increased after incubation with CAP at the dose of 1000, 2000, and 4000 a.u. (Figure 7A). In the normal group, exposure to CAP (1000, 2000, and 4000 a.u) has not changed the level of caspase-3 activation (Figure 7A). Also, results showed that Z-DEVD but not Z-IETD prevented CAP (2000 a.u) induced caspase-3 activation. These results suggest that CAP activates apoptosis signaling through the intrinsic mitochondrial pathway in the retinoblastoma group.

*CAP induced lysosomal damage*

In the retinoblastoma group, the results display a significant release of acridine orange ensued into the cytosolic fraction after incubation with CAP (1000, 2000, and 4000 a.u), indicating severe damage to the lysosomal membrane (Figure 7B).

## Discussion

RB is one of the intraocular (Retina) malignancies that occur in childhood and responsible for approximately 3% of malignancies in children. The survival rate of these patients in developing countries is lower than in developed countries, and the incidence is also higher (37-39). Various approaches have been used to treat RB, but their effectiveness is low (1, 3). So using a new approach can help to treat it (1, 5). One of the most important mechanisms involved in RB’s pathogenesis is the disruption of the apoptotic process, which has attracted many researchers (1, 5). In this study, the induction of oxidative stress, lysosomal damage, and apoptosis in RB mitochondria was investigated through exposure to CAP. Today, many studies have shown that CAP has been used to treat a variety of cancers. The generation of ROS is one of the most important mechanisms in which CAP causes death in cancer cells (11, 13, and 40).

At first, the SDH assay showed that exposure to CAP had reduced only the mitochondrial SDH activity in the RB group. The goal of cancer therapy is to eliminate cancer cells without effect on normal cells (12). Further, the results showed that exposure to plasma increased the production of free radicals in the mitochondria of the RB group. An increase in intracellular oxidative stress in cancer cells leads to their more vulnerability to oxidative damage (9). Also, research has shown that some compounds with an increase in ROS generation can be useful in cancer therapy. It is widely described that ROS cause cell death, especially apoptotic death (41-43). The induction of apoptosis in malignant cells can be considered as one of the new approaches to cancer therapy (44). 

In brain cancer cells, CAP induced cell death via extrinsic/death receptor pathways, including Fas, DR4, DR5, and TNFR1 receptors (45). In the melanoma cell lines, CAP induced apoptosis through Sestrin2 expression and activated downstream iNOS, p38 MAPK, and Fas signaling (46). It has been shown that CAP induces apoptosis by an increase in the level of free radicals (ROS/NOS) and subsequent activation of caspases (caspase 3/7) (47). In addition, reports indicated CAP induced apoptosis through an increase in ROS level, collapse in the MMP, and release of cytochrome c (48). Research has shown that the mitochondrial respiratory chain (especially complexes I and III) plays an important role in generating ROS in the cell (49, 50). It has also been shown that mitochondria play a key role in the apoptotic process. Hence, mitochondria are considered as a new therapeutic target in cancer therapy (22, 23).

It has been shown that the CAP depolarizes the mitochondrial membrane and release cytochrome c through inducing oxidative stress (12). The results showed that exposure to the CAP causes collapse at the MMP in the RB group. This result is consistent with the previous study has shown that exposure to CAP could damage the mitochondrial membrane (12). In this research, the mitochondrial swelling and cytochrome c release as subsequent events following mitochondrial permeability transition (MPT) were examined. The results showed that exposure to the CAP causes swelling in the mitochondria in the RB group. An increase in ROS levels has been associated with a decrease in mitochondrial GSH content and an increase in LPO. The results show that CAP with an effect on mitochondria can increase ROS generation and weaken antioxidant status in the cells. The results showed that exposure to CAP resulted in cytochrome c release from mitochondria in the RB group. This process can selectively trigger the induction of apoptosis signaling in mitochondria isolated from the retinoblastoma group. 

In this study, results indicated that the CAP caused damage to the lysosomal membrane in RB cells. According to Haber- Weiss reaction, it has been shown that H_2_O_2_ as a free radical has the potential to cross the lysosomal lipid membrane and generate a highly reactive hydroxyl radical (OH•). Additionally, studies have shown that lysosomes may play a role in the induction of apoptosis by releasing a protease enzyme (51, 52). The mitochondrial and lysosomal pathways are also linked together via Bcl-2 family proteins (53, 54). Finally, CAP at all applied concentrations selectively induced caspase-3 activation in the RB group. Caspase-3, as a final mediator of apoptosis, play an important role in the process of cell death (23).

**Table 1:** Technical parameters of the cold atmospheric plasma (CAP) settings applied to mitochondria

**Cold atmospheric plasma (CAP) parameters**	
Voltage	4 kV
Frequency	40 kHz
Time	30, 60, and 120 sec

## Conclusion

In conclusion, the results suggest that plasma through the effect on the mitochondrial respiratory chain and lysosomes increases ROS generation only in cancerous but not normal ocular mitochondria. ROS can increase the content of LPO and reduce the antioxidant defense capacity. Furthermore, the results provide evidence that mitochondrial and lysosomal targeting are the critical mechanisms by which CAP could potentially and selectively induce apoptosis in RB cells through caspase-3 activation. Finally, our result underlined CAP as a promising therapeutic candidate against RB and recommends the process for further research.
